# Cerebral arterial flow dynamics during systole and diastole phases in young and older healthy adults

**DOI:** 10.1186/s12987-023-00467-8

**Published:** 2023-09-13

**Authors:** Kimi Piedad Owashi, Cyrille Capel, Olivier Balédent

**Affiliations:** 1 CHIMERE UR 7516, Jules Verne University of Picardy, Amiens, France; 2 Medical Image Processing Department, Amiens Picardy University Medical Center, Amiens, France; 3 Neurosurgery Department, Amiens Picardy University Medical Center, Amiens, France

**Keywords:** Cerebral arterial flow, Phase-contrast MRI, Aging, Systole, Diastole

## Abstract

**Background:**

Since arterial flow is the leading actor in neuro-fluids flow dynamics, it might be interesting to assess whether it is meaningful to study the arterial flow waveform in more detail and whether this provides new important information. Few studies have focused on determining the influence of heart rate variation over time on the arterial flow curve. Therefore, this study aimed to evaluate cerebral arterial flow waveforms at extracranial and intracranial compartments in young and elderly healthy adults, also considering systole and diastole phases.

**Methods:**

Cine phase-contrast magnetic resonance imaging (CINE-PC MRI) was performed on twenty-eight healthy young volunteers (HYV) and twenty healthy elderly volunteers (HEV) to measure arterial blood flows at the extracranial and intracranial planes. A semi-automated protocol using MATLAB scripts was implemented to identify the main representative points in the arterial flow waveforms. Representative arterial profiles were estimated for each group. Moreover, the effects of age and sex on flow times, amplitude-related parameters, and parameters related to systole and diastole phases were evaluated at the extracranial and intracranial compartments. Student’s t-test or Wilcoxon’s test (depending on the normality of the distribution) was used to detect significant differences.

**Results:**

In HYVs, significant differences were observed between extracranial and intracranial levels in parameters related to the AP1 amplitude. Besides the detected differences in pulsatility index (extracranial: 0.92 ± 0.20 vs. 1.28 ± 0.33; intracranial: 0.79 ± 0.15 vs. 1.14 ± 0.18, *p* < .001) and average flow (715 ± 136 vs. 607 ± 125 ml/min, p = .008) between HYV and HEV, differences in the amplitude value of the arterial flow profile feature points were also noted. Contrary to systole duration (HYV: 360 ± 29 ms; HEV: 364 ± 47 ms), diastole duration presented higher inter-individual variability in both populations (HYV: 472 ± 145 ms; HEV: 456 ± 106 ms). Our results also showed that, with age, it is mainly the diastolic phase that changes. Although no significant differences in duration were observed between the two populations, the mean flow value in the diastolic phase was significantly lower in HEV (extracranial: 628 ± 128 vs. 457 ± 111 ml/min; intracranial: 599 ± 121 vs. 473 ± 100 ml/min, *p* < .001). No significant differences were observed in the arterial flow parameters evaluated between females and males in either HYV or HEV.

**Conclusion:**

Our study provides a novel contribution on the influence of the cardiac cycle phases on cerebral arterial flow. The main contribution in this study concerns the identification of age-related alterations in cerebral blood flow, which occur mainly during the diastolic phase. Specifically, we observed that mean flow significantly decreases with age during diastole, whereas mean flow during systole is consistent.

## Introduction

Intracranial pressure (ICP) pulse changes during the cardiac cycle result from the interplay between rapid intracranial arterial inflow and its compensatory mechanisms: the intracranial compliance, the cerebrospinal fluid (CSF) flow oscillations, and the venous outflow [1, 2].

ICP monitoring, initially introduced by Lundberg [3], has been used for diagnosing and managing different brain diseases, such as hydrocephalus, idiopathic intracranial hypertension, traumatic brain injury, and cerebral haemorrhages [4–6]. Currently, only the mean ICP value is considered in clinical practice; however, a growing number of researchers are interested in studying the shape of the ICP pulse waveform [7–9] to better understand the pathology’s origin [10]. Typically, the ICP pulse is composed of three peaks, P1, P2, and P3, which in healthy cases, P1 predominates in amplitude over P2 and P3 [11]. In pathological cases, P2 starts to increase above P1, which is related to decreased cerebrospinal compliance [7]. Earlier researchers [11, 12] postulated that these three peaks were consistent with the analogous components of the arterial pulse wave: systolic peak (P1), tidal peak (P2), and aortic valve closure peak (P3). Other studies [13, 14] suggested that P1 primarily reflected the pulsations of intracranial arteries and that P3 reflected venous pulsations. Hamilton et al. [8] found a significative positive correlation between the width of P2 and the aqueduct stroke volume in suspected normal pressure hydrocephalus patients. Based on their findings, the authors speculate that capillary-phase brain expansion or the pulsations in the ventricular choroid plexus are possible drivers of ventricular CSF pulsatile movement and may contribute to the P2 morphology.

Furthermore, Balédent et al. [15] were among the first to model intracranial pressure based on Marmarou’s monoexponential function and intracranial volume change (calculated from transcranial net flows of blood and CSF during one cardiac cycle acquired by phase-contrast MRI). They identified four periods in the ICP pulse: p1 induced by the systolic arterial inflow; p2 where the CSF and the venous flows are set in motion to reduce ICP until a pressure equilibrium is achieved between extracranial and intracranial compartments; p3 established as the brain equilibrium period (BEP), representing a particular time in the cardiac cycle when cerebral blood volume is stable; and p4 corresponding to a decrease in pressure whereas the arterial flow also decreases.

Thus, many theories have been proposed to better understand the ICP pulse morphology; however, the physiological mechanisms underlying the ICP changes over a cardiac cycle remain unclear. We believe that similarly to intracranial volume dynamics, the peaks P1, P2, and P3 are mainly related to the initial rapid intracranial arterial inflow and with the compensatory mechanisms of the CSF flow oscillations and the venous outflow, respectively.

Pulsatile ICP signals are mainly influenced by the arterial inflow pulse overlapped with the respiratory cycle since, as demonstrated in a recent study using real-time phase-contrast MRI [16], cerebral arterial flow is affected by free breathing. Since the arterial flow is the leading actor in neuro-fluids flow interactions, it might be interesting to assess whether studying the arterial flow waveform in more detail is meaningful. It could also be relevant for studying the ICP waveform, particularly the P1 peak.

It is well known that large central arteries dilate and stiffen with ageing, leading to increased cerebral blood flow (CBF) pulsatility [17], raised systolic blood pressure, and a gradual decrease in diastolic blood pressure [18]. Consequently, arterial compliance is reduced, resulting in a steepening of the diastolic pressure contour decay and a reduction in the diastolic pressure amplitude [19]. Steady-state CBF is also shown to progressively decreases in normal ageing, reflecting a reduced cerebral metabolic rate [17, 20]. Significant differences have also been reported [21–23] between the male and female subgroups in cerebral arterial flow.

Although several studies [24–27] have analysed arterial flow waveform and compared flow amplitude-related parameters in healthy or pathological, young or elderly populations, fewer studies have focused on assessing the influence of heart rate on the arterial flow curve. The cardiac cycle includes two essential phases: the systole, representing the initial and rapid ejection of arterial blood, and the diastole, which constitutes the relaxation period. The heart rate is the major determinant affecting diastole and systole duration; consequently, studying cerebral arterial flow considering systole and diastole phases could provide new important information.

The objective of this study was to evaluate cerebral arterial flow waveforms at extracranial and intracranial compartments in young and elderly healthy adults using cine phase-contrast magnetic resonance imaging (CINE-PC MRI). Firstly, to estimate a representative arterial profile in each group and compare their shape morphology. And secondly, to assess the effects of age and sex on flow times and amplitude-related parameters, as well as on parameters related to systole and diastole phases.

## Materials and methods

### Study populations

The studied population comprised twenty-eight healthy young volunteers (HYV) and twenty healthy elderly volunteers (HEV).

HYV (14 women and 14 men; mean ± SD age: 25 ± 4; age range: 19–32), with no neurological, psychiatric, or severe general disease, alcoholism, or abnormalities detected by a clinical MRI exam, were considered for the present study. HEV (14 women and 6 men; mean ± SD age: 72 ± 6 years; age range: 60–84 years) were all recruited to the Neurology department of the Amiens university hospital and examined by a neurologist with ten years of experience to exclude seizures, transient neurological deficits, or gait disorders. Elderly volunteers underwent a neuropsychological screening with a Mini-Mental State Examination (MMSE); only subjects with a score above 26/30 were included. Subjects with relevant cerebral neurological disease (cerebrovascular accident, meningoencephalitis, tumour) or relevant cerebrovascular risk factors, except arterial hypertension controlled by medication, were excluded. Participants with cerebral ventricular enlargement were also excluded from the study.

HYV and HEV underwent an MRI examination, which lasted about 30 min. The study was conducted in accordance with the principles outlined in the Declaration of Helsinki and approved by the local Ethics Review Committee. All subjects signed a written informed consent before participating in the study protocol.

### PC-MRI acquisition

**Fig. 1:**
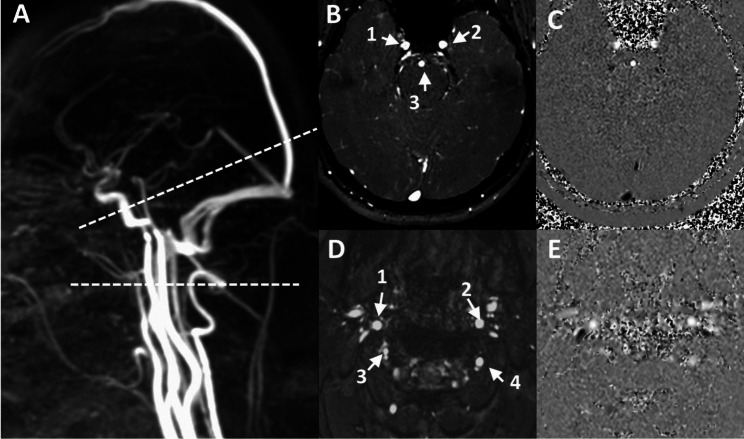
Extracranial and intracranial acquisition imaging. Sagittal 3D phase-contrast angiography **(A)** was used as reference to set the different acquisition planes perpendicular to the flow direction of blood inflow. **B** and **C (D** and **E)** represent the amplitude and phase images of the intracranial (extracranial) plane, respectively. For the intracranial plane, 1: right internal carotid artery, 2: left internal carotid artery, 3: basilar artery. For the extracranial plane, 1: right internal carotid artery, 2: left internal carotid artery, 3: right vertebral artery, 4: left vertebral artery

Participants were examined in the supine position, using a clinical 3T MRI system. Sagittal 3D phase-contrast angiography (Fig. 1A) was used as reference to position the intracranial (below the Circle of Willis) and extracranial (first cervical levels) acquisition planes for cerebral arterial blood flow measurements using the CINE-PC sequence. The acquisition planes were selected perpendicular to the presumed direction of the blood inflow. The intracranial plane (Fig. 1B&C) contains the left and right internal carotid arteries (ICAs_intra), and the basilar artery (BA); the extracranial plane (Fig. 1D&E) includes the left and right internal carotid arteries (ICAs_extra), as well as the left and right vertebral arteries (VAs).

Table 1 shows the main settings used for the CINE-PC sequence in both populations. During the acquisition of CINE-PC images, a finger plethysmograph was used for retrospective cardiac gating. After 50–115 s of acquisition (depending on the subject’s heart rate), all the data were reconstructed into 32 phase-contrast images representing the blood flow rate variation within an average cardiac cycle (CC).

### Flow measurements

Arterial blood flow measurements were performed using in-house software, “Flow” [28]. The software performs a semi-automated segmentation of the blood flows in the PC-MRI images. The segmentation algorithm is based on temporal pixel intensity variation compared to the cardiac cycle frequency. For all segmented pixels in the region of interest (ROI), 32 fluid velocity values are calculated over the CC, averaged and multiplied by the ROI area to finally calculate the total flow rate through the ROI.

**Table 1:** Main parameters used for the CINE-PC sequence in HYV and HEV.

Parameters	
VENC (mm/s)	600–800
FOV (cm^2^)	14 ^2^
Resolution (mm^2^)	0.5^2^
Thickness (mm)	2–5
Flip angle (degree)	30
SENSE	1.5
TE (ms)	6.6
TR (ms)	10.9

VENC: velocity encoding; FOV: field-of-view; SENSE: sensitivity encoding; TE: echo time; TR: repetition time

**Fig. 2:**
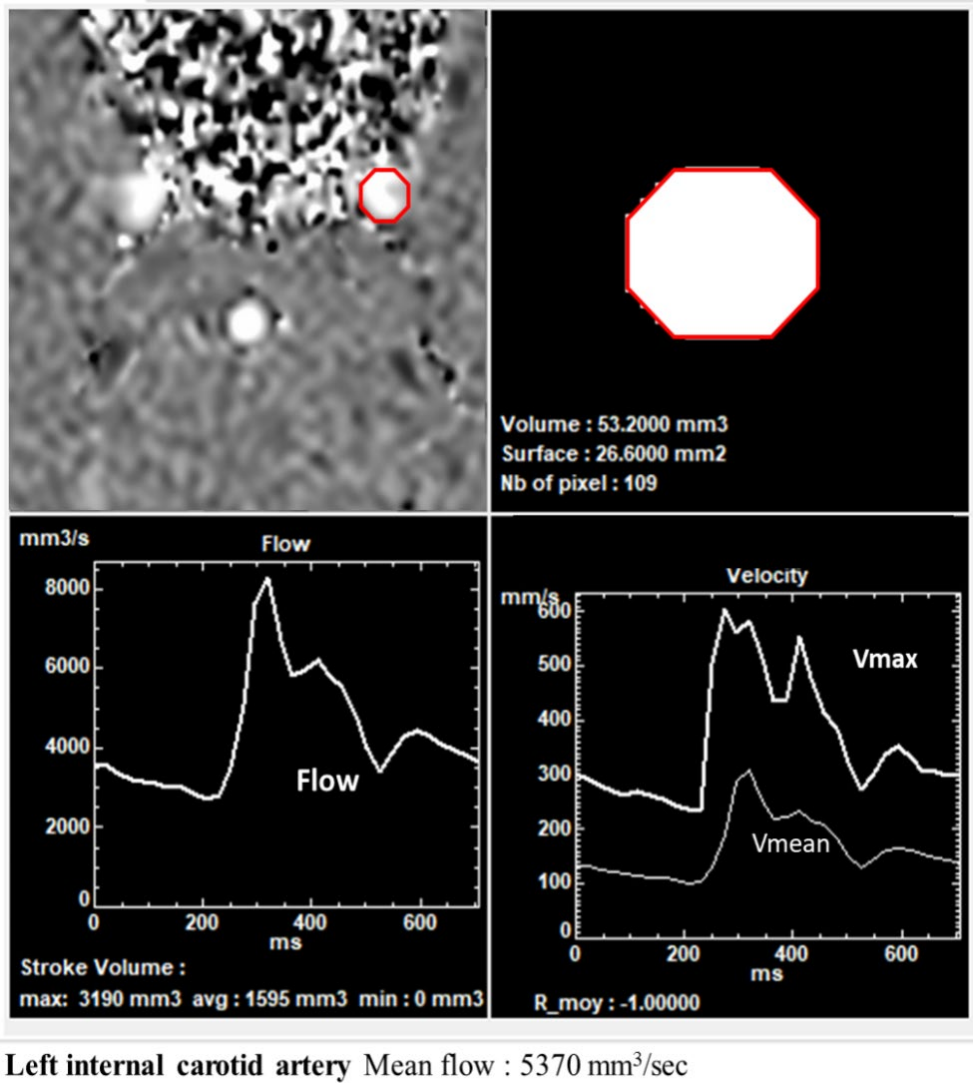
Example of flow quantification of the left internal carotid artery at the intracranial level using the “Flow” software. The software performs a semi-automatic segmentation and calculates the ROI area and the fluid velocity over the cardiac cycle. Subsequently, the software extracts the average flow waveform from the selected vessel

Thus, the software can extract the arterial flow waveform in the selected vessel (Fig. 2). The location and size of the ROI are assumed to be constant throughout the CC. Refer to previous studies [24, 28] for more details on PC-MRI image processing.

Total extracranial cerebral arterial blood flow (Qa_total_extra) was calculated by adding the two ICAs_extra and the two VAs flows. Similarly, total intracranial cerebral arterial blood flow (Qa_total_intra) consisted of the sum of the two ICAs_intra and the BA flows.

### Identification of the main representative points of the arterial flow waveform

**Fig. 3:**
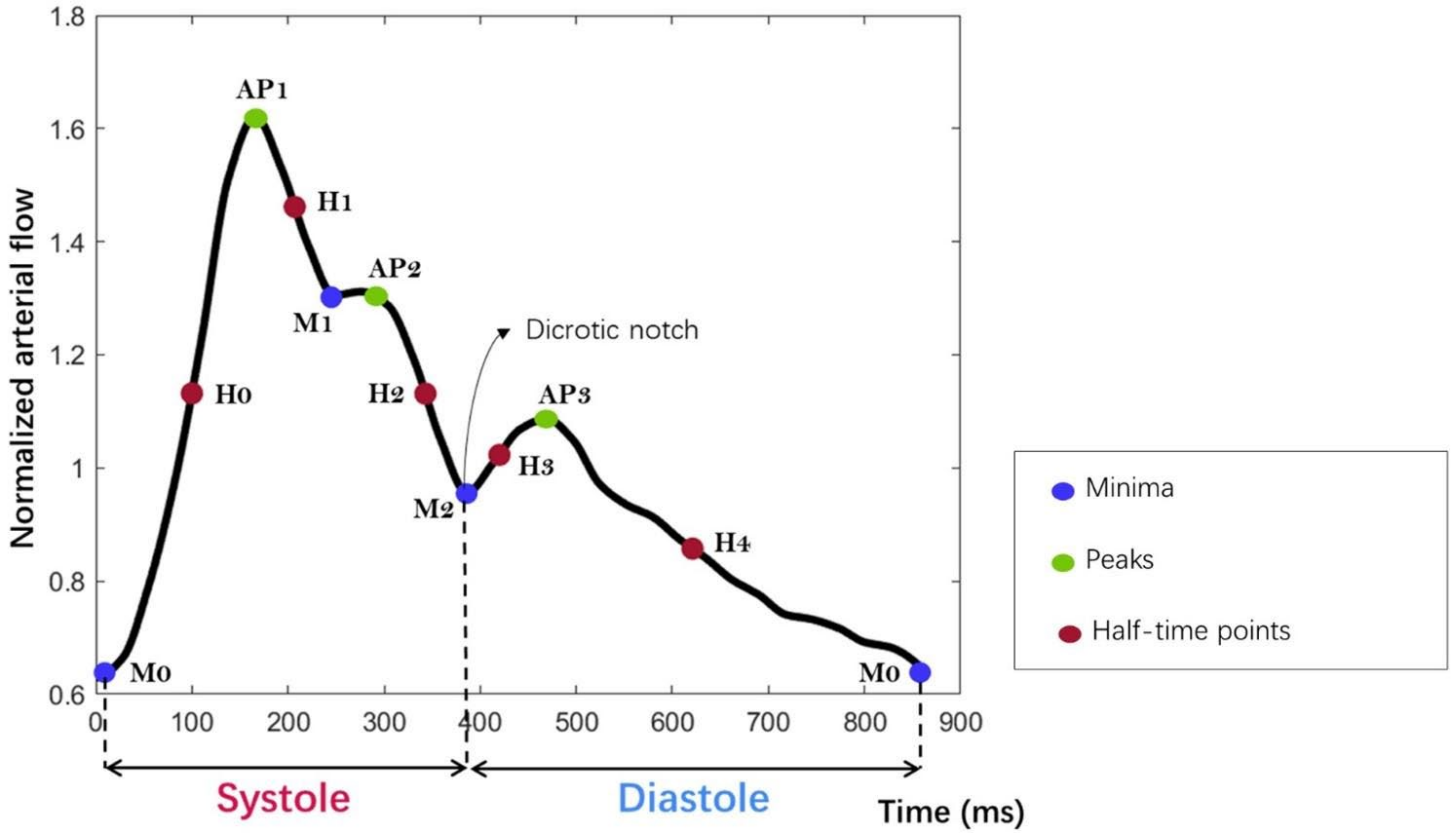
Main representative points of the arterial flow waveform. Example of an arterial flow curve normalized by its cycle-average flow value. M0, M1, and M2 (in blue) denote the three main minima observed in the arterial flow waveform (AFW). AP1, AP2, and AP3 (in green) indicate the three main peaks at the AFW. H0, H1, H2, H3 and H4 (in red) are the half-time points between M0-AP1, AP1-M1, AP2-M2, M2-AP3 and AP3-M0, respectively. M2, known as the dicrotic notch, connotes the end of systole and the beginning of diastole. Points identification and calculation of systole and diastole duration were performed using scripts in MATLAB (version 2019b, Mathworks, Natick, WA, USA)

**Fig. 4:**
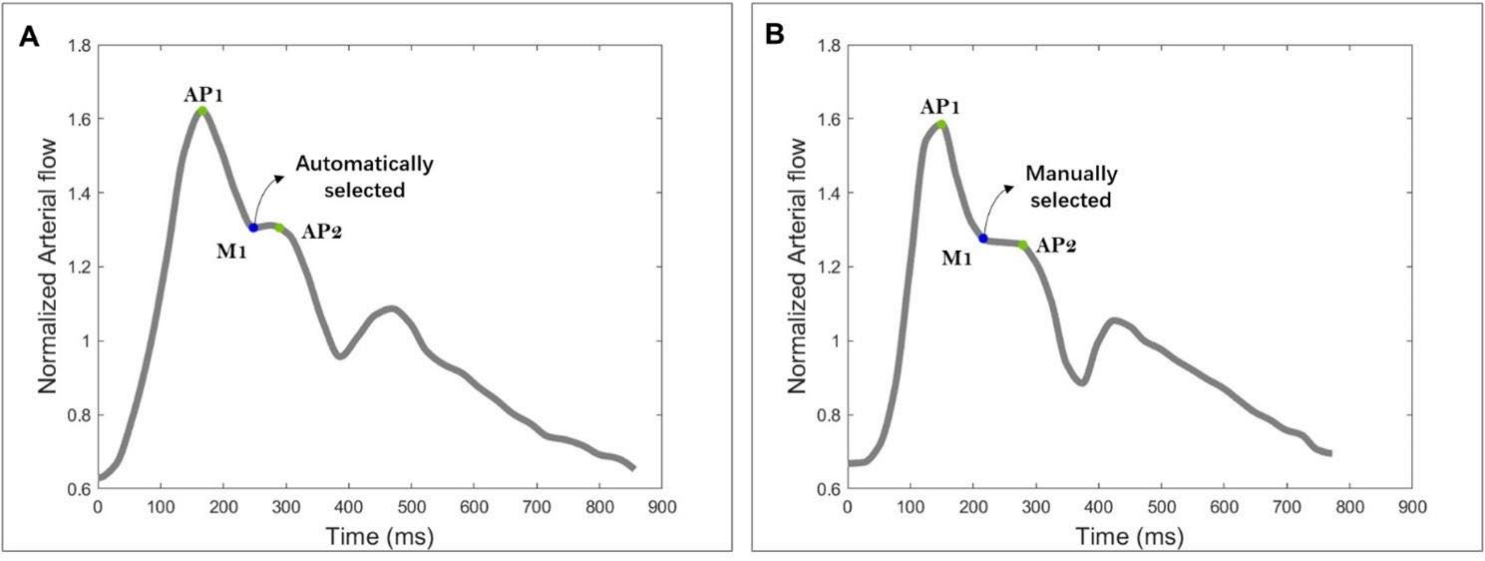
Examples of arterial flow waveforms (AFW) in two subjects. **(A)** The behaviour of the AFW enables the automatic selection of point M1, as it demonstrates a sign change in the first derivative accompanied by a change in the curve’s concavity. **(B)** The AFW monotonically decreases between AP1 and AP2, indicating that the curve does not increase between these points. Consequently, no sign change was observed in the first derivative at point M1, which necessitated manual selection

Inspired by [26], we propose a semi-automated protocol using MATLAB scripts (version 2019b, Mathworks, Natick, WA, USA) to identify the main representative points in the total arterial flow waveform (AFW), and characterize its shape in HYV and HEV at the extracranial and intracranial levels. This protocol consists, first, in normalizing the AFW with respect to the cycle-average flow rate value. Then, from the normalized and cubically interpolated AFW, the algorithm identifies the curve’s global minimum (M0) and maximum peak (AP1). Complementary minima and peaks (M1, AP2, M2, AP3) were also determined by identifying, starting from AP1, the sign change of the flow curve’s first derivative. Indeed, the sign of the first derivative is negative when the flow is decreasing and positive when it is increasing. Thus, the sign change will indicate the beginning of an increasing or decreasing section of the curve, representing, respectively, a minimum or a peak in the signal. The half-time points between the minima and peaks (H0, H1, H2, H3, H4) were also estimated and located in the AFW. These eleven main points were identified in the AFWs of all the HYV and HEV. Figure 3 illustrates an example of a normalized and cubically interpolated AFW with the main points identified and located on the curve. In some instances, the arterial curve exhibits a monotonically decreasing pattern between AP1 and AP2, i.e., the AFW does not increase and remains free of concavity changes. As a result, the curve does not generate a sign change in the first derivative at M1, requiring the manual selection of this point. Figure 4 provides an illustrative example of the AFW in two different subjects. In one case, M1 was successfully selected automatically, while in the other case, manual selection was necessary due to the monotonically decreasing behaviour of the curve.

All the AFWs in each group were synchronized relative to H0 in order to compensate for any variability in the cardiac gating between subjects. Subsequently, it was possible to construct a representative arterial flow curve for HYV and HEV at the extracranial and intracranial levels by fitting the mean values of the eleven points previously identified in each AFW. Then, these representative AFWs were multiplied by the total mean flow values of the corresponding population for visual comparison of arterial flows in HYV and HEV extra- and intracranially.

### Amplitude-related parameters of the arterial flow waveform

**Fig. 5:**
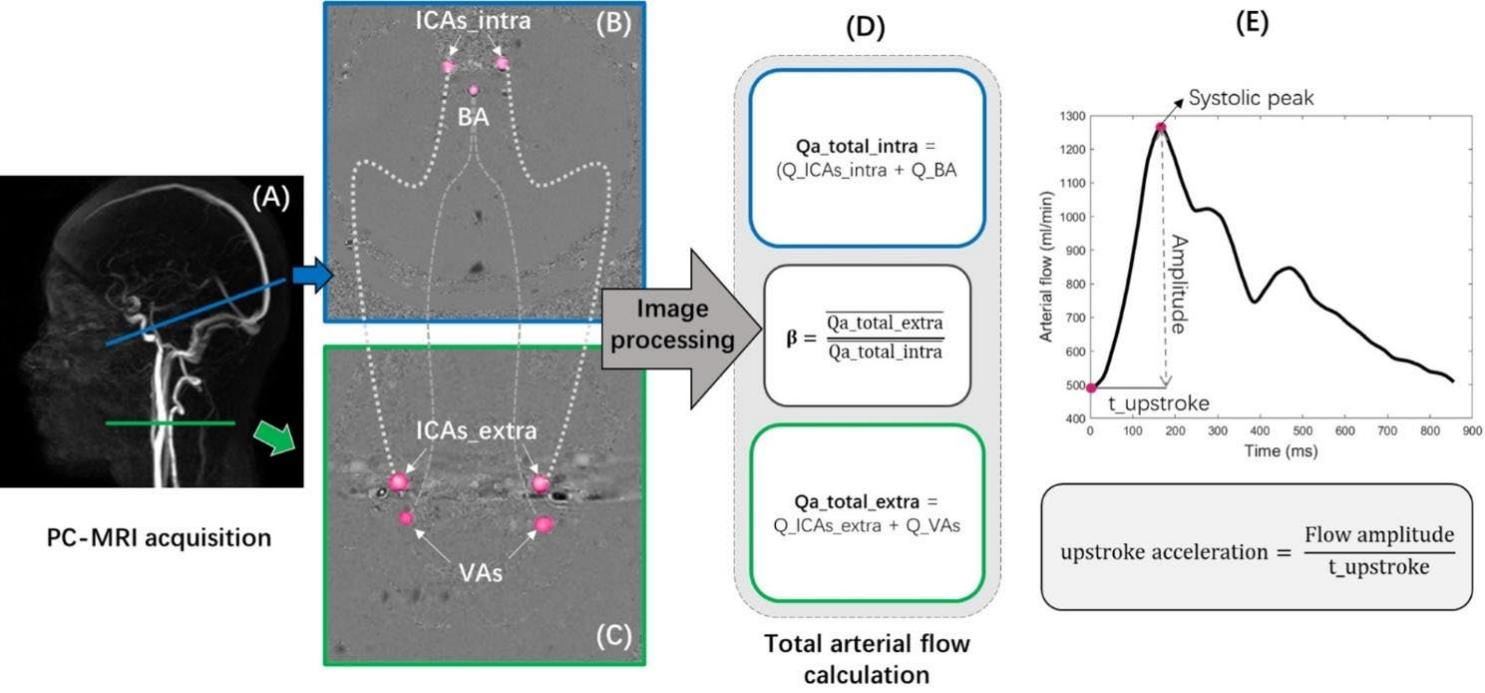
Flow chart of image acquisition and calculation of amplitude-related parameters of the arterial flow curve. **(A)** Sagittal three-dimensional angiography is used as a reference to position the intracranial (blue line) and extracranial (green line) acquisition planes. **(B)** Phase-contrast images in the intracranial acquisition plane were used to quantify flow in the right and left internal carotid arteries (ICAs_intra) and the basilar artery (BA). **(C)** Phase-contrast images in the extracranial acquisition plane were used to quantify flow in the right and left internal carotid arteries (ICAs_extra) and the right and left vertebral arteries (VAs). **(D)** The sum of the flows in the ICAs_extra (Q_ICAs_extra) and VAs (Q_VAs) is equal to the total extracranial arterial flow (Qa_total_extra). Similarly, the sum of the flows in the ICAs_intra (Q_ICAs_intra) and BA (Q_BA) is equal to the total intracranial arterial flow (Qa_total_intra). The parameter β is calculated by dividing the mean value of the arterial flow at the extracranial level by that at the intracranial level. **(E)** An example of the arterial flow curve shape in a healthy young volunteer. The upstroke acceleration parameter is estimated from the flow amplitude and the time between the baseline and the systolic peak

The Qa_total_extra and Qa_total_intra curves from each HYV and HEV were compared with regard to some parameters related to the AFW. A parameter β was calculated by dividing the cycle-average flow value at the extracranial level by the cycle-average flow value at the intracranial level (Fig. 5D). Parameters such as the pulsatility index (PI) and damping factor (DF), investigated in several arterial flow studies [24, 29–31], were also estimated:

$$PI = \frac{{{\rm{Flow}}\,{\rm{amplitude}}}}{{{\rm{Mean}}\,{\rm{flow}}}},$$

$$\begin{array}{*{20}{c}}{\begin{array}{*{20}{c}}{DF = \frac{{{\rm{P}}{{\rm{I}}_{{\rm{extra}}}}}}{{{\rm{P}}{{\rm{I}}_{{\rm{intra}}}}}},}\end{array}}\end{array}$$

where the flow amplitude was calculated by subtracting the maximum and minimum flow values. The amplitude value of the peaks AP1 and AP2 were measured and compared. Similarly, the amplitude values for the subtraction between AP1 and M0 (AP1-M0) and AP2 and M0 (AP2-M0) were also calculated.

To describe the rapid upstroke of arterial inflow, we measured the steep rise time from the baseline to the systolic peak (t_upstroke) in the arterial flow curve. We also introduced the notion of ‘upstroke acceleration’ by dividing the flow amplitude by the t_upstroke (Fig. 5E).

### Systolic and diastolic phases of the arterial flow waveform

Based on the representative points of the AFW previously identified, the durations of the systolic and diastolic phases were measured. Point M2, known as the dicrotic notch, is mainly associated with the closure of the aortic valve and represents the end of systole and the start of diastole. Therefore, the time elapsed between M0 to M2 indicates the duration of the systolic phase, while the time elapsed between M2 to M0 indicates the duration of the diastolic phases (Fig. 3). The systole-average and diastole-average flow values for each HYV and HEV were also estimated and compared.

### Statistical analysis

The statistical analyses were performed with MATLAB software scripts (version 2019b, Mathworks, Natick, WA, USA). Descriptive statistics were expressed as the mean ± SD. The Shapiro-Wilk test was used to determine whether the data were normally distributed. Student’s t-test or Wilcoxon’s test (depending on the normality of the distribution) was used to detect differences between HYV and HEV and paired tests assessed differences between extracranial and intracranial flow parameters of each group. The results were considered significant at *p* < .05.

## Results

### Arterial flow waveforms in HYV and HEV at the extracranial and intracranial level

**Fig. 6:**
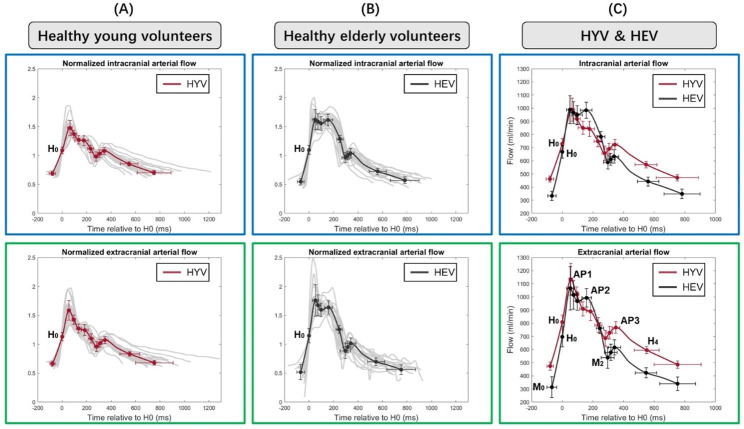
Representative arterial flow waveforms in young and elderly healthy population at extracranial and intracranial level. The total arterial flow curves are aligned relative to H0. The figure shows in light grey the total extracranial (green box) and intracranial (blue box) arterial flow curves of the twenty-eight healthy young volunteers **(A)** and the twenty healthy elderly volunteers **(B)**. We obtained the representative arterial flow waveforms in HYV (in red, **A**) and HEV (in black, **B**) by fitting the eleven average points identified in the entire population. The representative waveforms were multiplied by the total mean flow value of the corresponding population to compare the arterial profiles in HYV and HEV **(C)**. Note that the amplitude scale in C is different from A&B. The arterial waveforms in C are not normalized

The normalised total arterial flow curves of each younger and older volunteer were aligned relative to H0 and presented in light grey in Fig. 6A and B, respectively. We obtained the representative extra- and intracranial arterial profiles for HYV (in red) and HEV (in black) from the eleven main points identified semi-automatically on each of these curves. The mean and standard deviation (in amplitude and time) of these points are indicated on the representative curves. These extra- and intracranial waveforms were multiplied by the total mean flow value of the corresponding population to compare arterial flows in HYV and HEV (Fig. 6C).

As shown in Fig. 6, the shape of the representative arterial flow curves was preserved at the extra- and intracranial level for each population. In all these arterial profiles, the onset of systole can be clearly identified by the initial steep rise in flow. The dicrotic notch indicating the start of diastole is also easy to recognise. We also noticed that the points located at the diastolic tail showed higher variability in terms of time in all cases compared to the systolic points. Figure 6C demonstrates a significantly larger difference between AP1 and AP2 amplitudes for HYV than for HEV, extracranially (*p* < .001) and intracranially (*p* < .001). Moreover, Fig. 6C also clearly shows that the amplitudes of points H0 and AP3 are higher in HYV than in HEV; conversely, the amplitude of AP2 is greater in HEV than in HYV.

### Comparison of extracranial and intracranial flow parameters within HYV and HEV

The results of the comparison of arterial flow-related parameters at extra- and intracranial levels in HYV versus HEV are summarized in Table 2. The HYV and HEV population groups were further categorized into two subgroups based on the sex of the volunteers. Table 2 also summarizes the results and comparisons across all these subgroups.

The heart rate (HYV:72 ± 12 bpm versus HEV:71 ± 11 bpm; p = .78), β correction factor (HYV:1.08 ± 0.11 versus HEV: 1.00 ± 0.16; p = .08), and damping factor (HYV: 1.11 ± 0.25 versus HEV:1.15 ± 0.3; p = .73) do not differ significantly between the young and elderly populations.

For HYVs, significant differences were observed between the extracranial and intracranial values of AP1 (extra: 1125 ± 201 ml/min versus intra: 982 ± 184 ml/min; *p* < .001), AP1-M0 (extra: 649 ± 144 ml/min versus intra: 521 ± 117 ml/min; *p* < .001) and PI (extra: 0.92 ± 0.20 versus intra: 0.79 ± 0.15; *p* < .001). When examining HYVs categorised by gender, significant differences were observed between the extracranial and intracranial values of PI (*p* < .001) and upstroke acceleration (*p* < .001) in HYV males but not in females. Conversely, the mean flow between extra- and intracranial compartments was significantly different (p = .003) in HYV females but not in males. HEVs also exhibited significant differences between the extracranial and intracranial values of AP1-M0 (p = .007) and PI (p = .003).

The mean value of the extracranial flow was significantly higher in HYV (715 ± 136 ml/min) compared to HEV (607 ± 125 ml/min; p = .008); however, the estimated mean intracranial flow value showed no significant difference between HYV (669 ± 133 ml/min) and HEV (610 ± 125 ml/min; p = .1). The amplitude values of AP1, extracranially and intracranially, did not show significant differences between HYV and HEV, similarly for the extracranial AP2 value. Nevertheless, when comparing the AP1-M0, AP2-M0, and PI, HEV demonstrated significantly higher values at both extracranial and intracranial compartments than HYV (*p* < .001; p = .02 for extracranial AP1-M0).

Extra- and intracranial upstroke times in HEV (extra: 120 ± 45 ms and intra: 118 ± 44 ms) were significantly shorter than in HYV (extra: 134 ± 30 ms, p = .03; and intra: 144 ± 39 ms, p = .003). Inversely, extra- and intracranial upstroke accelerations were markedly higher in HEV (extra: 121 ± 52 ml/s^2^ and intra: 108 ± 38 ml/s^2^) than in HYV (extra: 85 ± 29 ml/s^2^, p = .013; and intra: 66 ± 27 ml/s^2^, *p* < .001).

Additionally, regarding the gender-based comparison in Table 2, no significant differences were observed in the arterial flow parameters evaluated between females and males in either HYV or HEV.

**Table 2:** Comparison of intracranial and extracranial arterial flow-related parameters in young and elderly healthy adults

Parameters	HYV	HEV	p-value	HYV male	HYV female	p-value	HEV male	HEV female	p-value
n	28	20		14	14		6	14	
Age (yr)	25 ± 4	72 ± 6		26 ± 4	25 ± 3		72 ± 7	72 ± 6	
Heart rate (bpm)	72 ± 12	71 ± 11	0.78	69 ± 13	75 ± 11	0.38	70 ± 11	73 ± 11	0.63
β	1.08 ± 0.11	1 ± 0.16	0.08	1.06 ± 0.13	1.09 ± 0.09	0.57	1.01 ± 0.22	1 ± 0.1	0.88
Damping factor	1.11 ± 0.25	1.15 ± 0.3	0.73	1.19 ± 0.28	1.03 ± 0.18	0.22	1.21 ± 0.38	1.1 ± 0.3	0.59
E. mean flow (ml/min)	715 ± 136	607 ± 125	0.008^**^	669 ± 125	761 ± 136	0.07	621 ± 156	601 ± 116	0.74
I. mean flow (ml/min)	669 ± 133	610 ± 125	0.1	635 ± 124	704 ± 137	0.17	619 ± 124	606 ± 113	0.83
	**p = .002** ^**^	**p = .89**		**p = .13**	**p = .003** ^**^		**p = .96**	**p = .81**	
E. AP1 (ml/min)	1125 ± 201	1076 ± 194	0.4	1100 ± 179	1149 ± 225	0.53	1169 ± 240	1036 ± 165	0.17
I. AP1 (ml/min)	982 ± 184	1021 ± 174	0.5	948 ± 150	1016 ± 213	0.33	1080 ± 202	995 ± 162	0.34
	**p = < 0.001** ^***^	**p = .15**		**p = < 0.001** ^***^	**p = < 0.001** ^***^		**p = .38**	**p = .30**	
E. AP2 (ml/min)	889 ± 176	991 ± 205	0.07	832 ± 191	946 ± 145	0.09	1019 ± 251	979 ± 165	0.70
I. AP2 (ml/min)	798 ± 202	1010 ± 271	0.003^**^	772 ± 216	823 ± 192	0.51	1026 ± 338	1004 ± 252	0.87
	**p = .01** ^*^	**p = .79**		**p = .26**	**p = .01** ^*^		**p = .97**	**p = .72**	
E. AP1-M0 (ml/min)	649 ± 144	760 ± 178	0.02*	655 ± 118	643 ± 171	0.82	863 ± 230	716 ± 138	0.09
I. AP1-M0 (ml/min)	521 ± 117	686 ± 137	< 0.001^***^	502 ± 81	539 ± 146	0.35	744 ± 174	661 ± 115	0.22
	**p = < 0.001** ^***^	**p = .007** ^**^		**p = < 0.001** ^***^	**p = .003** ^**^		**p = .14**	**p = .02** ^*^	
E. AP2-M0 (ml/min)	413 ± 92	674 ± 163	< 0.001^***^	387 ± 112	439 ± 60	0.14	713 ± 183	657 ± 157	0.49
I. AP2-M0 (ml/min)	361 ± 104	666 ± 195	< 0.001^***^	342 ± 108	380 ± 100	0.35	682 ± 262	659 ± 170	0.82
	**p = .004** ^**^	**p = .87**		**p = .08**	**p = .03** ^*^		**p = .84**	**p = .95**	
E. PI	0.92 ± 0.20	1.28 ± 0.33	< 0.001^***^	1 ± 0.21	0.84 ± 0.18	0.11	1.44 ± 0.41	1.22 ± 0.28	0.23
I. PI	0.79 ± 0.15	1.14 ± 0.18	< 0.001^***^	0.81 ± 0.17	0.76 ± 0.14	0.63	1.22 ± 0.23	1.1 ± 0.16	0.19
	**p = < 0.001** ^***^	**p = .003** ^**^		**p = < 0.001** ^***^	**p = .05**		**p = .04** ^*^	**p = .02** ^*^	
E. upstroke time (ms)	134 ± 30	120 ± 45	0.03^*^	137 ± 24	131 ± 36	0.62	113 ± 40	123 ± 47	0.39
I. upstroke time (ms)	144 ± 39	118 ± 44	0.003^**^	147 ± 35	141 ± 43	0.66	124 ± 70	115 ± 30	0.65
	**p = .24**	**p = .78**		**p = .37**	**p = .42**		**p = .44**	**p = .59**	
E. acceleration (ml/s^2^)	85 ± 29	121 ± 52	0.013^*^	82 ± 20	88 ± 36	0.60	142 ± 63	112 ± 47	0.24
I. acceleration (ml/s^2^)	66 ± 27	108 ± 38	< 0.001^***^	60 ± 15	72 ± 35	0.26	120 ± 48	102 ± 34	0.37
	**p = .002** ^**^	**p = .14**		**p = < 0.001** ^***^	**p = .11**		**p = .23**	**p = .38**	

Values are means ± SD. All data were tested for the normality of their distribution and then compared with a Student’s t-test or Wilcoxon’s test. (*: *p* < .05; **: *p* < .01; ***: *p* < .001). The p-values in bold correspond to the comparison of each group’s parameters at the extracranial and intracranial levels. E.: extracranial; I.: intracranial; PI: pulsatility index

### Systole and diastole phases

**Fig. 7:**
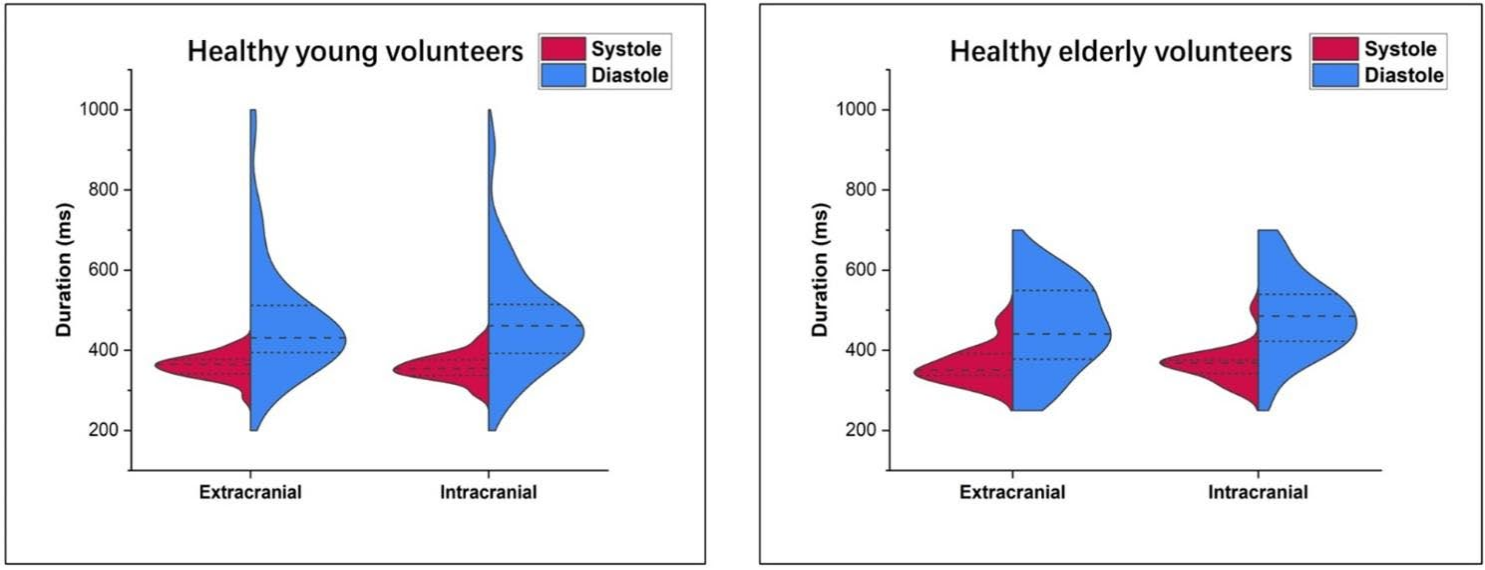
Mirrored density plot of systole (red) and diastole (blue) duration at extra- and intracranial levels in HYV (left) and HEV (right). The three lines in the density diagram represent the interquartile range and the median. Higher variability was observed in the duration of the diastolic phase. However, the systolic phase duration is less variable in both HYV and HEV at the extra- and intracranial levels

Concerning the duration of the systolic and diastolic phases of the arterial flow curve, it was measured by calculating the elapsed time between M0-M2 and M2-M0 respectively. The mean ± standard deviation of the participant’s systolic and diastolic phase durations is presented in Table 3. The results showed no significant differences between extra- and intracranial phase durations in either HYV or HEV. Likewise, the duration of systolic and diastolic phases did not differ considerably between each study population. However, Fig. 7 clearly illustrates a higher variability in the duration of the diastolic phase at both extracranial and intracranial levels in HYV and HEV compared to the systolic phase duration. In contrast, the systolic phase duration is less variable in both HYV and HEV at both acquisition levels.

**Table 3:** **Comparison of intracranial and extracranial parameters related to diastole and systole flow in young and elderly healthy adults**

Parameters	HYV	HEV	p-value	HYV male	HYV female	p-value	HEV male	HEV female	p-value
n	28	20		14	14		6	14	
E. CC duration (ms)	832 ± 158 *(100%)*	820 ± 111 *(100%)*	0.79	876 ± 193	788 ± 102	0.35	860 ± 122	803 ± 106	0.31
I. CC duration (ms)	831 ± 150 *(100%)*	850 ± 118 *(100%)*	0.29	869 ± 176	793 ± 113	0.39	941 ± 108	811 ± 101	0.02^*^
**Systole**									
E. duration (ms)	360 ± 29 *(* *44%* *)*	364 ± 47 *(* *45%* *)*	0.71	363 ± 25	358 ± 34	0.68	361 ± 29	365 ± 54	0.87
I. duration (ms)	357 ± 32 *(* *44%* *)*	365 ± 43 *(* *43%* *)*	0.49	359 ± 31	355 ± 33	0.77	387 ± 64	356 ± 29	0.15
	**p = .59**	**p = .61**		**p = .64**	**p = .78**		**p = .25**	**p = .47**	
E. mean flow (ml/min)	843 ± 153	816 ± 172	0.57	797 ± 142	889 ± 154	0.11	852 ± 207	800 ± 161	0.55
I. mean flow (ml/min)	774 ± 154	809 ± 143	0.43	736 ± 138	812 ± 165	0.20	839 ± 171	796 ± 135	0.55
	p = < 0.001***	**p = .84**		p = .03*	p = .002**		**p = .87**	**p = .91**	
**Diastole**									
E. duration (ms)	472 ± 145 *(* *56%* *)*	456 ± 106 *(* *55%* *)*	0.85	513 ± 178	431 ± 90	0.36	498 ± 134	438 ± 92	0.26
I. duration (ms)	474 ± 131 *(* *56%* *)*	485 ± 99 *(* *57%* *)*	0.47	510 ± 151	437 ± 100	0.21	554 ± 111	455 ± 80	0.04^*^
	**p = .54**	**p = .06**		**p = .91**	**p = .45**		p = .02*	**p = .38**	
E. mean flow (ml/min)	628 ± 128	457 ± 111	< 0.001^***^	587 ± 120	669 ± 126	0.09	472 ± 143	451 ± 99	0.70
I. mean flow (ml/min)	599 ± 121	473 ± 100	< 0.001^***^	571 ± 118	627 ± 123	0.23	482 ± 115	470 ± 97	0.81
	p = .03*	**p = .36**		**p = .42**	p = .02*		**p = .81**	**p = .34**	

Values are means ± SD. All data were tested for the normality of their distribution and then compared with a Student’s t-test or Wilcoxon’s test. (*: *p* < .05; **: *p* < .01; ***: *p* < .001). The p-values in bold correspond to the comparison of each group’s parameters at the extracranial and intracranial levels. In the second and third columns, the duration was also defined as a percentage of cardiac cycle. E.: extracranial; I.: intracranial

**Fig. 8:**
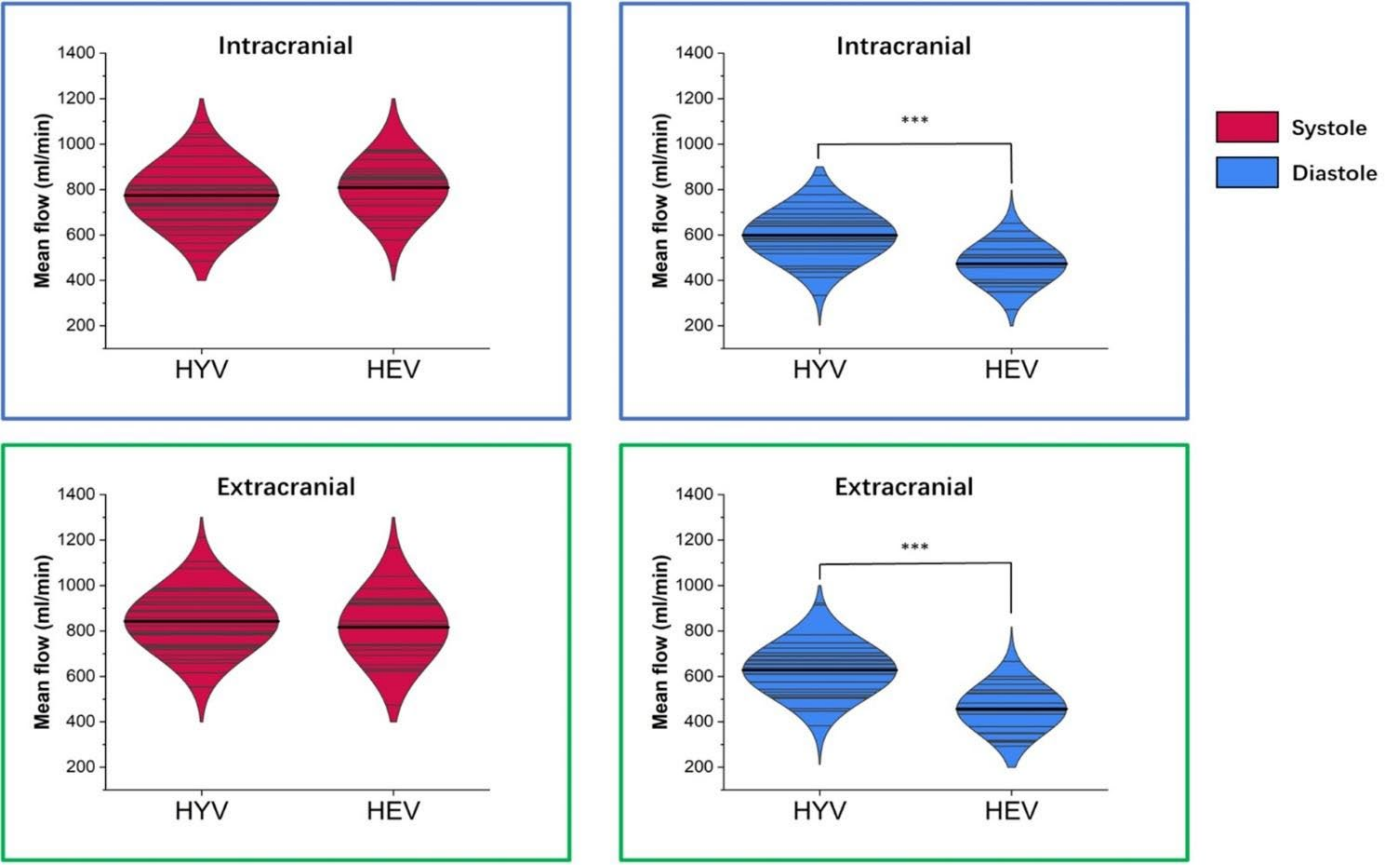
Distribution of the values of extracranial (green box) and intracranial (blue box) mean flow in systole (red) and diastole (blue) for HYV and HEV. Each thin grey line represents the mean flow value for each participant, and the bold line represents the total average value of the corresponding group. Systole-average flow values at extra- and intracranial levels were comparable in HYV and HEV. In contrast, diastole-average flow values at both levels were significantly higher in HYV than in HEV. (***: *p* < .001). HYV: healthy young volunteer; HEV: healthy elderly volunteer

The data in Table 3 show that, with regard to systole, the mean flow value did not exhibit a significant difference between extracranial and intracranial levels in HEV, while there was an important difference in HYV (*p < .001*) . Also, when comparing the systole-average flow value in HYV and HEV, it did not present a significant difference. However, interestingly, the diastole-average flow value was significantly higher in HYV than in HEV at both extracranial (HYV: 628 ± 128 ml/min versus HEV: 457 ± 111 ml/min; *p* < .001) and intracranial (HYV: 599 ± 121 ml/min versus HEV: 473 ± 100 ml/min; *p* < .001) levels (Fig. 8).

Additionally, regarding the gender-based comparison of the arterial parameters in the systolic and diastolic phases (Table 3), no significant differences were observed, except for the duration of diastole, which was significantly shorter (p = .04) in females HEV at the intracranial plane. Regarding the gender-based comparison between age groups, while both females and males presented a decrease in the mean flow during the diastole phase with age, the difference between young and older women was found to be significant (*p* < .001).

**Fig. 9:**
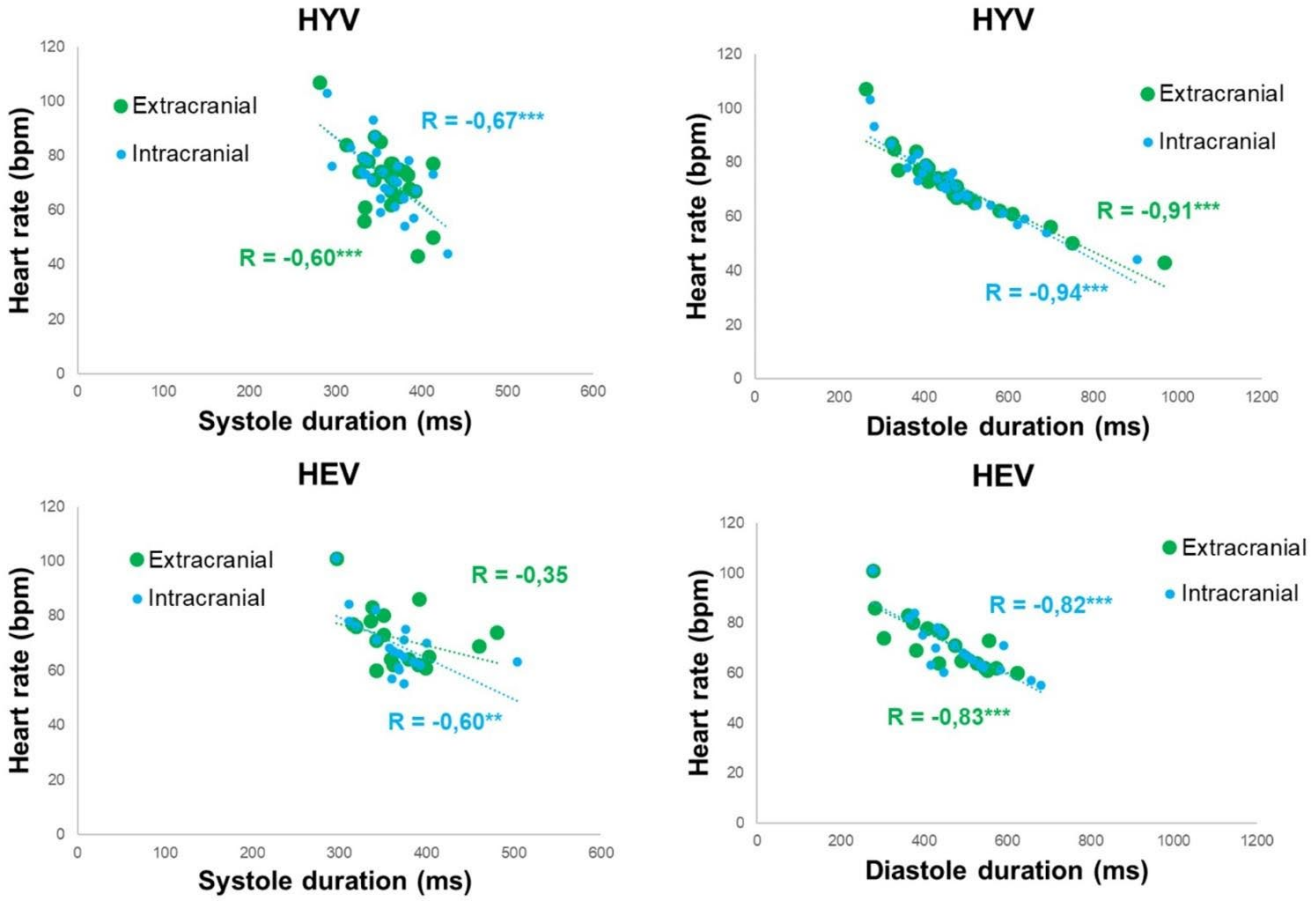
Correlation between the duration of systole (left) and diastole (right) phases with heart rate assessed at the extracranial (green) and intracranial (blue) planes in HYV (top) and HEV (bottom). R is Spearman’s correlation coefficient (**: *p* < .01, ***: *p* < .001). HYV: healthy young volunteer; HEV: healthy elderly volunteer

Systole duration and heart rate showed an important correlation in HYV and HEV (for intracranial measurements). Moreover, Fig. 9 also illustrates a stronger and significant correlation (*p* < .001) mainly between diastole duration and heart rate in both HYV (extra- R: -0.91 and intra- R: -0.94) and HEV (extra- R: -0.83 and intra- R: -0.82).

## Discussion

This paper evaluated cerebral arterial flows extracted from PC-MRI images to compare the effect of (i) age, (ii) individual’s heart rate variation over time, and (iii) sex on extracranial and intracranial arterial haemodynamics. Identifying the main representative points in the cerebral arterial waveforms allowed, in addition to obtaining representative flow profiles, to elucidate arterial flow differences during systole and diastole.

### Effect of normal ageing in arterial flow profiles

The arterial flow waveform, under normal conditions, is represented by an anacrotic branch (i.e., an ascending pulse) followed by a descending dicrotic branch (i.e., double pulse). The anacrotic branch corresponds to the steep rise from M0 to AP1 peak (upstroke period). On the other hand, the dicrotic branch represents the progressive decrease in blood flow during late systole and diastole. The AP2 peak, seen in the dicrotic branch, is thought to represent the arrival of the reflected wave from local peripheral vasculature after ventricular ejection [32]. The dicrotic notch, M2, coincides with aortic valve closure and the beginning of diastole. And AP3 is associated with diastolic wave reflections [33].

Firstly, as observed in Fig. 6, the arterial flow profiles preserved their extracranial and intracranial waveform in the two study populations. Moreover, Fig. 6 illustrates that the most remarkable differences between HYV and HEV are the higher amplitude of AP2 and the lower amplitude of AP3 found in older adults’ waveforms. The age-related increase in arterial wall stiffness may contribute to a higher effect (in speed and magnitude) of reflected waves during systole, leading to an earlier and higher flow and, thus, a rise in peak AP2. Reduced AP3 may be related to a diminished Windkessel effect. With age, the elastic arteries become less compliant, and their reservoir function is impaired [34], reducing the volume of blood ejected during diastole and, thus, the diastolic wave reflections (AP3). These findings are similar to Hoi et al. [35] study, which also reported a stronger AP2 and significantly lower AP3 in older adults’ ICA flow. Contrary to Hoi et al., the present study compared HEV and HYV arterial profiles scaled to the corresponding population cycle-average flow, revealing also greater amplitude of M0, H0, M2, and H4 in HYV.

These representative arterial profiles not only facilitate the analysis and understanding of the curve morphology in various healthy or pathological populations, but also may serve as inputs for computational modelling aimed at simulating the dynamics of the craniospinal system. The arterial inflow, being of primary importance in cerebral dynamics, plays a crucial role in these computational models.

### Extracranial and intracranial compartments in arterial flow

The paired vertebral arteries, besides the basilar artery supply, also provide blood to the cerebellum arteries, which is not included in the measurement approach described in this study. Balédent et al. [15] reported that about 13.5% of extracranial arterial blood volume reached the cerebellum in healthy young adults. The factor β presented in our study represents the difference between cervical and intracranial mean flow values, which provides an estimation of the volume of blood supplying the cerebellum’s arterial vascularisation (i.e., about 8% for HYV and 0.5% for HEV). The percentage of blood volume reaching the cerebellum is not negligible, especially in younger adults; still, we hypothesise that cerebellar vascularisation does not influence the haemodynamics between the extracranial and intracranial compartments and is therefore not taken into account.

One of the interests of this study was to compare the extra- and intracranial arterial flow profiles. Therefore, instead of assessing the representative flow rate waveform in each artery as Ford et al. [26] proposed, we added the flows of the major arteries to evaluate the total representative cerebral arterial flow at both levels. As concluded in [26], the two vertebral and internal carotid arteries presented little qualitative difference between the shapes of the respective arterial flow waveforms, both within and between healthy subjects, with lower mean flow values in VAs than in ICAs. Our findings are comparable to those of Ford et al., the flow curves in both VAs and ICAs_extra (and, in our case, also in BA and ICAs_intra) showed highly similar waveforms with insignificant temporal shifts between them. Moreover, their reported total cycle-averaged arterial flow value at the cervical level for HYV, 731 ml/min, was consistent with our result of 715 ± 136 ml/min. Thus, since the flow profiles of both extra- and intracranial arteries were closely similar and synchronised, considering the total cerebral arterial flow at each level does not introduce significant modifications for assessing the craniospinal arterial haemodynamics.

### Effect of normal ageing in flow-related parameters

Several studies [36–38] have reported a significant decrease in arterial blood flow with normal ageing, reflecting a reduced cerebral metabolic rate [17]. Our present results also revealed a considerable diminution in the mean arterial flow with ageing, especially at the extracranial level. The PI and the flow amplitude, as already indicated in previous studies [17, 24, 31, 32] showed remarkable differences between the populations, being significantly higher in HEV than in HYV due to the known increase of arterial wall stiffness with age [20].

The upstroke acceleration of the arterial inflow reflects the rate at which the velocity of cerebral arterial flow changes in terms of volume per second squared. As shown in Table 2, the upstroke acceleration is significantly higher in HEV, indicating that the system is under greater stress compared to HYV. The difference in upstroke acceleration may also be attributed to the loss of compliance and increased stiffness of the arteries with age. Some studies have used the arterial blood-flow acceleration term to predict significant arterial stenosis or estimate the overall impact of peripheral artery disease [39], predict intracranial aneurisms [40], or characterize newborn infants [41]. To our knowledge, the analysis of upstroke acceleration is not commonly explored in the study of cerebral arterial hemodynamics. However, given that the upstroke acceleration of the cerebral arterial inflow represents the initial trigger in the craniospinal system hemo-hydrodynamic, it could provide valuable insights for studying arterial dynamics in brain pathologies. Furthermore, considering the upstroke acceleration in the analysis of ICP pulse morphology could also provide a further understanding of the underlying mechanisms.

The HYV and HEV groups showed no apparent influence of sex on the main arterial flow-related parameters. Alwatban et al. [22] reported a significantly lower PI in females between 18 and 30 years old at the middle cerebral artery. However, in our measures, there were no significant gender differences in the PI measured extracranially and intracranially in HYV.

The relationship between sex and cerebral blood flow were studied extensively in the past [42–44] and most authors observe higher mean flow in women than in men. Our findings align with these studies in the HYV population, showing a higher mean flow value in females, although not statistically significant. However, this pattern was not observed in the HEV population. Additionally, a significant difference in mean arterial flow was observed between young and older healthy females, suggesting a more substantial decrease in mean flow with age in females.

When comparing females and males separately regarding age, some significant differences were observed in one sex but not the other. This suggests that sex can indeed have an impact on the results. However, based on the findings of this study, we believe that the differences in arterial flow profiles are influenced mainly by age rather than sex.

### Systole and diastole phases

The cardiac cycle is divided into two essential phases: systole and diastole. Systole is characterised as a phase of robust contraction responsible for the onset of rapid ejection of blood into the arteries, reflecting an initial substantial increase in cerebral arterial flow and its progressive decrease. During systole, arteries walls stretch to accommodate extra blood. On the other hand, diastole represents a more passive, relaxed condition, which coincides with a slight increase in cerebral arterial flow due to the extra blood ejection from the arterial wall.

Several findings were observed in the present study concerning systole and diastole flow parameters. Firstly, as shown in Fig. 7, systole duration was similar across all HYV and HEV, while diastole duration presented higher inter-individual variability in both populations. Indeed, diastole duration revealed a significantly negative correlation with heart rate (Fig. 9). For example, regarding the extracranial flow, the healthy young adult with the highest heart rate (107 bpm) presented the lowest diastole duration (∼264 ms), even lower than its systole duration (∼ 282 ms). In contrast, the healthy young adult with the lowest heart rate (43 bpm) showed a markedly longer diastole duration (∼ 970 ms), while the systole duration (∼396 ms) was not so far from the previous case. The same pattern was observed in older adults, suggesting that the diastole is adapted to preserve systole duration in normal ranges. Theoretically, it is known that, under standard conditions, systole and diastole represent approximately one-third and two-thirds, respectively, of the cardiac cycle. Nevertheless, our results show that this is only sometimes the case and that even the duration of diastole can be shorter than systole, depending on the subject’s heart rate. Hence, if it is desired to normalise the arterial flow curve in time, it is recommended to extend or reduce the diastolic duration rather than stretch or compress the entire waveform in order to conserve systole timings.

Secondly, our results show that, with age, it is mainly the diastolic phase that changes. Although no significant differences in time were observed, the mean flow value was significantly lower in HEV, as clearly observed in the bold average line in Fig. 8. These findings confirmed that, under normal conditions, the diastolic phase plays an important role in maintaining optimal systolic function (ensuring normal or desired values in terms of flow amplitude and timing) despite inter-individual variation in heart rate or age and what this implies in arteries’ mechanical properties.

When examining females and males separately in relation to age, a significant decrease in mean flow during the diastole phase was observed in females, both extracranially and intracranially. However, it is worth noting that the HEV group consisted of only 6 males and 14 females, which may have impacted the ability to detect significant differences. Further analysis and exploration may be necessary with a more homogenous group, notably for HEV, to determine the influence of sex in normal ageing.

Previous studies [45–47] on cardiac physiology and imaging have consistently shown that the duration of the diastole is more significantly influenced by the heart rate compared to the duration of systole. Although systole and diastole durations are well-known in cardiac physiology studies, this information is not commonly considered when studying cerebral arterial flow. Usually, cerebral arterial flow is studied by analyzing the entire curve as a whole in terms of the percentage of the cardiac cycle. However, dividing the curve into two phases, systole and diastole, and analyzing them separately can provide valuable and new information. To the best of our knowledge, there are currently no studies that specifically propose studying cerebral arterial flow in this manner. This approach may offer new insights into cerebral blood flow dynamics and its relationship to physiological and pathological conditions.

The intracranial pressure is also driven by the cardiac cycle, and the P1 peak (in ICP pulse) is mainly related to systolic arterial flow. Therefore, volume and time information related to arterial inflow represents the initial conditions of ICP pulse, and the results of the present study could indeed be relevant for the analysis of ICP waveforms. The perturbation of AP2 peak amplitude (in arterial flow curve) with age, mainly due to physiological modifications in the arteries, could have repercussions on ICP, generating morphological differences in pulse waveforms. Likewise, changes in mean arterial flow present with age, especially during the diastolic phase, could have an impact on CSF oscillation and, notably, on venous outflow haemodynamics.

Since ICP measurement is only ethically acceptable with clinical indications, reference values and ICP waveforms still need to be discovered. A previous study [48] analysed a population of children and adults as close to “normal” as possible to examine if age affects ICP. This study reported an apparent decrease in ICP with age, which may be partly associated with the age-related differences observed in arterial flow profiles.

In contrast, in some pathological conditions related to CSF disorders, which have been further studied, ICP appears to increase, and P1 (in ICP pulse) begins to lose strength in the pressure profile. The explanation behind this phenomenon is still unclear, and we believe that the analysis of arterial compartments but also venous and CSF compartments could help to elucidate the mechanisms underlying ICP variations. Therefore, these results may represent the basis for further arterial flow studies in patients with brain pathologies in order to provide new information for a better understanding of the ICP pulse profile.

### Limitations

One limitation of this work concerns the number of healthy young and elderly volunteers included in this study. In fact, arterial flow values are highly dispersed over the populations, and therefore, a larger sample size of volunteers is needed to reduce the impact on our results. It has been shown that arterial flow parameters measured in the carotid arteries are partly sex and body-size dependent; however, significant differences were observed in external but not internal carotids [35]. In the present study, we did not consider the effects of blood pressure or height on the arterial flow dynamic. Nevertheless, we are interested in evaluating cerebral arterial flow, and these parameters apparently would not significantly influence our results.

The presence of air produces a high susceptibility to artefacts, particularly in regions close to the paranasal sinuses there are significant field inhomogeneities that can distort the image [49]. Acquisitions at the intracranial level were obtained close to the nasal cavity, which could produce high susceptibility to image artefacts indeed. However, in this study, the artifacts caused by air were relatively minor, and their impact on the accuracy of the blood flow measurements was minimal.

In some cases, the algorithm used for this study failed to automatically identify the point M1 of the arterial flow waveform, necessitating manual selection. This limitation becomes more evident in studies with larger datasets. More advanced algorithms using artificial intelligence techniques could overcome this. These new algorithms aim to automatically identify the representative points of the arterial waveform by optimizing the minimum error between the AFW and the average arterial profile of the study population. This approach will improve the efficiency and accuracy of identifying the points in individual subjects.

## Conclusion

This study aimed to assess the impact of age, individual’s heart rate variation over time, and sex on extracranial and intracranial arterial parameters and profiles by evaluating total cerebral arterial flows derived from PC-MRI images.

Our study provides a novel contribution on the influence of the cardiac cycle phases on cerebral arterial flow, which, to the best of our knowledge, has not been extensively explored before. Our results showed that, despite variations in heart rate between subjects, both in young and aged populations, the systole phase duration of the cerebral arterial flow waveform was similar across all the HYVs and HEVs, suggesting that the duration of the diastole phase is adapted according to the subject’s heart rate.

Our results also revealed that, with age, the difference in cerebral blood flow is mainly due to the diastolic phase. Thus, mean flow decreases with age during diastole, whereas mean flow during systole is consistent.

The results of the present study provide valuable reference values of arterial flow in young and older healthy adults and add to the current understanding of the complex dynamics of arterial flow that may serve as a basis for further studies in patients with brain pathologies.

## Data Availability

Dicom data cannot be provided due to privacy laws. De-identified processed data can be provided upon reasonable request. To obtain the post-processing software and its user manual, please contact Dr. Olivier Balédent.

## List of abbreviations

AFW: Arterial flow waveform
BA: Basilar artery
CC: Cardiac cycle
CSF: Cerebrospinal fluid
DF: Damping factor
FOV: Field-of-view
HEV: Healthy elderly volunteers
HYV: Healthy young volunteers
ICA: Internal carotid artery
ICP: Intracranial pressure
PC-MRI: Phase-contrast magnetic resonance imaging
PI: Pulsatility index
ROI: Region of interest
SENSE: Sensitivity encoding
TE: Echo time
TR: Repetition time
VA: Vertebral artery
VENC: Velocity encoding
